# Family dementia caregivers with suicidal ideation improve with mentalizing imagery therapy: Results from a pilot study

**DOI:** 10.1016/j.jadr.2024.100721

**Published:** 2024-01-09

**Authors:** Saira Madarasmi, Paulina Gutierrez-Ramirez, Nader Barsoum, Sreya Banerjee, Liliana Ramirez Gomez, Maria Melero-Dominguez, Laura N. Gitlin, Aderonke Pederson, Richard T. Liu, Felipe A. Jain

**Affiliations:** a Department of Psychiatry, Depression Clinical and Research Program, Massachusetts General Hospital, Boston, MA, United States; b Harvard Medical School, Boston, MA, United States; c Department of Neurology, Massachusetts General Hospital, Boston, MA, United States; d College of Nursing and Health Professions, Drexel University, Philadelphia, PA, United States; e Harvard-Massachusetts Institute of Technology (MIT) Division of Health Sciences and Technology, Boston, MA, United States

**Keywords:** Family caregivers, Dementia, Mindfulness, Depression, Suicidal ideation, Guided imagery

## Abstract

**Background:**

Family caregivers of persons living with dementia often experience increased depression and suicidal ideation (SI). However, the feasibility and impact of therapies on caregiver SI has remained largely unexplored. Mentalizing imagery therapy (MIT) helps reduce psychological symptoms through mindfulness and guided imagery. This pilot study examined the feasibility of participation by caregivers with SI in a randomized controlled trial (RCT) of MIT versus a psychosocial support group (SG), and the respective impact of group on SI, depression, and secondary outcomes.

**Methods:**

A secondary analysis of data from an RCT of 4-week MIT or SG for caregivers (*n* = 46) was performed, identifying SI (*n* = 23) and non-SI (*n* = 23) cohorts. Group attendance and home practice were compared between cohorts. In the SI cohort (total *n* = 23, MIT *n* = 11, SG *n* = 12), group differences in SI, depression, and secondary outcomes were evaluated post-group and at 4-month follow-up.

**Results:**

Attendance in both groups and home practice in MIT were similar between SI and non-SI cohorts. In the SI cohort, MIT evinced greater improvements relative to SG in SI (*p*=.02) and depression (*p*=.02) post-group, and other secondary outcomes at follow-up.

**Limitations:**

Limitations include small sample size and single-item assessments of SI from validated depression rating scales.

**Conclusions:**

Participation in an RCT was feasible for caregivers with SI. MIT resulted in important benefits for SI and depression, while SG showed no acute SI benefit. The role of MIT in improving SI should be confirmed with adequately powered trials, as effective therapies to address caregiver SI are critical.

## 1. Introduction

Rates of suicide in the US have increased over the past 20 years, and suicidal ideation (SI) is an important suicide predictor (Turecki et al., 2019). People caring for family members living with dementia often experience elevated stress, depression and anxiety (Alzheimer’s Association, 2022), all of which have been associated with caregiver SI (Joling et al., 2018). A recent population-based study of more than 10, 000 adults in the United States found that nearly 40 % of unpaid caregivers reported passive SI, and more than 30 % seriously considered attempting suicide in the past month (Czeisler et al., 2021). Caregivers of those with cognitive decline had a threefold increased risk of SI relative to the general population (Czeisler et al., 2021), similar to other reports of family caregivers showing an elevated risk of SI (Joling et al., 2018).

Despite these striking findings, the feasibility and impact of therapeutic interventions for caregivers with SI have not been established. To our knowledge, most, if not all, large trials of therapeutic interventions to reduce depressive symptoms in caregivers have not reported on SI (Gaugler et al., 2018, 2008; Gitlin et al., 2003; Whitebird et al., 2013; Wilz et al., 2017). As a result, it is unclear to what extent behavioral interventions may help alleviate SI in this population.

Mentalizing imagery therapy (MIT) teaches guided imagery and mindfulness techniques to improve self- and other-understanding and aims to reduce negative psychological symptoms such as depression and anxiety (Jain and Fonagy, 2020). We recently published the first randomized, controlled trial (RCT) of MIT versus a psychoeducational support group (SG), which found that MIT yielded superior improvements in depressive symptoms, anxiety, stress, and mindfulness (Jain et al., 2022a). Moreover, functional magnetic resonance imaging performed before and after the group interventions in this RCT demonstrated strengthening of dorsolateral prefrontal cortex connectivity with an emotion regulation network in MIT but not SG (Jain et al., 2022a). This resting state connectivity increase was correlated with increases in mindfulness and reductions in depressive symptoms, indicating a possible mechanism of symptom improvement (Jain et al., 2022a).

Given the high rates of SI observed in the family caregiver population, in the current study we aimed to study the impact of MIT and SG on participants with SI. We performed a secondary analysis of data from the RCT to assess the feasibility of participation in the RCT by family caregivers with SI. We also aimed to identify patterns of change in SI, other psychological symptoms, and related outcomes, including depression, anxiety, perceived stress, caregiver burden, and mindfulness. We hypothesized that, while participation in both arms of the trial would be feasible, participants in MIT would evince greater improvements than SG in SI and other secondary outcomes.

## 2. Methods

### 2.1. Design

This secondary analysis aimed first to characterize the SI cohort (*n* = 23) versus the non-SI cohort (*n* = 23) on baseline measures including demographic and caregiving variables, and psychological symptoms including depression, anxiety, perceived stress, caregiver burden, and mindfulness. We then determined whether the feasibility and acceptability of participation in the trial between caregivers with SI versus those without SI was similar. Following establishment of feasibility and acceptability, for our second aim we focused solely on the SI cohort (total *n* = 23; MIT *n* = 11; SG *n* = 12). Analyses of the SI cohort aimed to determine whether credibility of therapy differed based on group assignment, and whether outcomes in the SI cohort differed between MIT and SG.

### 2.2. Participants

This analysis utilized data from a pilot RCT for caregivers of family members with dementia (*n* = 46) (NCT# 03092050; https://clinicaltrials.gov/ct2/show/NCT03092050). All procedures were performed in accordance with the Institutional Review Boards of the University of California, San Francisco, and Massachusetts General Hospital. Inclusion criteria were: (1) being the primary caregiver for a family member with dementia, (2) being age 40 and older, and (3) having English fluency. Age was restricted to 40 and older so as to achieve a sample of middle-aged and older adult caregivers who together comprise the largest population of family dementia caregivers. Exclusion criteria were: having (1) a primary psychiatric disorder other than unipolar depression, (2) cognitive impairment, (3) any unstable medical illness or planned procedure that would interfere with participation, (4) indication of violent tendencies or intent to harm their relative with dementia, or an open Adult Protective Services report on file, and (5) an existing meditation/imagery practice more than twice a week.

### 2.3. Procedures

Caregivers were block randomized into MIT (*n* = 24) or a psychosocial SG (*n* = 22). Each group of caregivers participated in weekly 2-hour meetings over 4 weeks. The MIT group learned and practiced mindfulness and guided imagery exercises. Exercises were designed to engage participants in the various facets of mentalization, focusing on understanding their own mental states and those of their family member with dementia (Jain et al., 2022a). Weekly exercises included mindful stretching, breathing-focused meditation, and guided imagery practices. Mindful stretching comprised slow extension and rotational movements of the limbs and trunk, while maintaining a present-focused attitude. Breathing-focused meditation included exercises to notice sensations of the body with a strong focus on the breath. Guided imagery practices included imagination of self and others, including switching perspectives to imagine how others might have perceived the participant in challenging situations. Guided imagery exercises also contained an explicit focus on interconnectedness of self and others. Please refer to our prior open access publication for further details of MIT exercises (Jain et al., 2021). Home practice exercises were assigned with a suggestion to complete them as many days as possible, and a goal of at least 4 to 5 times per week. The SG arm received a facilitated discussion group that utilized a problem-solving approach to address participant caregiving challenges and provided brief dementia psychoeducation. The SG was not assigned home practice exercises because the study aimed to compare MIT to a common community-based intervention for family caregivers rather than to study specific efficacy of MIT components. Participants completed self-rated psychological symptom questionnaires and clinician-rated depression at baseline, immediately post-group, and at a 4-month follow-up visit. Additionally, researchers obtained self-rated depressive symptoms and home practice logs for MIT exercises prior to the second, third, and fourth weekly group meetings. Caregivers were blinded to study groups and hypotheses, but outcomes assessors were not blinded.

From 6 weeks prior to study entry until the post-group assessment (approximately 10 weeks total), participants who were in treatment were prohibited from changing doses of antidepressants or frequency of psychotherapy. Those with elevated depressive symptoms at baseline were informed that they could benefit from evidence-based approaches to treat depression, but uniformly preferred to continue within the study. Post-group, those with persistent symptoms of low mood or anxiety were encouraged to receive evidence-based treatment.

### 2.4. Measures

Feasibility of participation in the RCT was measured by group attendance and dropout rate. Adherence in the MIT group was assessed by completion of the guided imagery and mindfulness practices assigned as homework (at least 4 times a week for 4 weeks). Credibility of therapy was measured using a Likert scale from 1 to 10 assessing logicality, confidence in success, likelihood of recommendation, and success in treating other kinds of stress (Borkovec and Nau, 1972). Standardized assessments for psychological assessments included the Quick Inventory of Depressive Symptomology – Self Report (QIDS; Rush et al. 2003), the Hamilton Depression Rating Scale (HAM-D; Hamilton 1960), the State-Trait Anxiety Inventory (STAI; Spielberger et al. 1970), the Caregiver Burden Scale (CBS; O’Rourke and Tuokko 2003), the Perceived Stress Scale (PSS; Cohen et al. 1983), and the Five Facet Mindfulness Questionnaire (FFMQ; Baer et al. 2008). Internal consistency as estimated by Cronbach’s α was calculated for all measures at all time points using package “ltm” in R (Rizopoulos, 2007), and was found to be acceptable: HAM-D baseline (T1)=0.82, post-group (T2)=0.84, 4-month follow-up (T3) =0.85; QIDS T1=0.82, T2=0.70, T3=0.82; STAI T1=0.94, T2=0.95, T3=0.95; PSS T1=0.89, T2=0.86, T3=0.89; CBS T1=0.92, T2=0.90, T3=0.85; FFMQ T1=0.93, T2=0.90, T3=0.93. A single item on the self-report depression measure, QIDS (#12, “Thoughts of Death or Suicide”), or clinician-rated HAM-D (#3, “Suicide: Covers Suicidal Tendencies and Plans”) was used to assess for presence of SI. At baseline, participants were considered to have SI if they scored at least a 1 on either measure, indicating passive (thoughts of wanting to die) or active (suicidal plan or intent) SI. SI was thus dichotomized into 0=no SI or 1=presence of SI.

### 2.5. Statistical analysis

The primary analysis of the parent trial, comparing the effects of MIT and SG across all participants, was previously reported (Jain et al., 2022a). The current analysis focused on differences between SI and non-SI cohorts at baseline and treatment outcomes only in the SI cohort. All statistical analyses were performed in R 4.2.2 (R Core Team, 2016). For the first aim, Fisher’s exact test and non-parametric *t*-tests were used to calculate baseline differences between SI (*n* = 23) and non-SI (*n* = 23) cohorts. Attendance and home practice data were evaluated with non-parametric *t*-tests between the SI and non-SI cohort.

For the second aim, participants with SI (*n* = 23) in the MIT (*n* = 11) and SG (*n* = 12) groups were identified for secondary analysis of credibility and symptom change. Total scores were calculated for all psychological questionnaires. Using package “nlme” in R (Pinheiro et al., 2023), Group by Time effects on psychological symptoms were assessed covarying for age and sex (consistent with the parent RCT (Jain et al., 2022a)) with mixed linear models across 3 time points: baseline, post-group, and 4-month follow-up. Effect sizes on symptom changes were estimated with Cohen’s *d*.

To assess for changes in SI in MIT (*n* = 11) and SG (*n* = 12), we determined whether there was a numeric change in SI on the QIDS or HAM-D at each time point. Worsened SI was defined as a higher score for the participant on the QIDS or HAM-D suicidality question; persistent SI as the same severity on the QIDS or HAM-D suicidality question; and improvement as a reduction in the QIDS or HAM-D suicidality question. When one measure stayed the same and the other showed change (improvement or worsening), the participant was categorized as having shown signs of change (Supplementary Table 1). There were no instances of opposing changes in SI (in which improvement was shown on one measure but worsening on the other). The number of participants experiencing worsened SI, persistent SI or improved SI was determined from baseline to post-group and baseline to 4-month follow-up, and Fisher’s exact test computed for group differences.

In the SI cohort (*n* = 23), there was missing data for outcomes analysis due to loss to 4-month follow-up of one participant in MIT and one in SG. These two participants were excluded from the analysis of SI change at the 4-month follow-up timepoint. Missingness in completion of secondary assessments (<10 % of all questionnaires) was accounted for by an intent-to-treat analysis in which all participants were included in mixed linear models.

In the SI cohort, to determine the time course of change in symptoms in MIT (*n* = 11) and SG (*n* = 12), we conducted post hoc, within-group, paired *t*-tests, between baseline and post-group, and baseline and 4-month assessments. Cohen’s d for within-group changes were calculated as (Mean of the change scores)/(Standard Deviation of the change scores).

## 3. Results

### 3.1. Aim 1: characterization of SI (n = 23) versus non-SI (n = 23) cohorts

#### 3.1.1. Association between baseline variables and SI

At baseline, QIDS (*p*=.005), HAM-D (*p*=.0001), STAI (*p*=.004) and PSS (*p*=.02) were greater for SI (*n* = 23) than non-SI (*n* = 23) participants. There was no difference, however, between groups for demographic variables, CBS, or FFMQ (*p*>.05 for all measures, please refer to online supplementary material for demographics table).

#### 3.1.2. Feasibility and acceptability between SI and non-SI participants

Group attendance was similar between the SI and non-SI cohorts (*p*=.9), with participants attending an average of 3 (SD 1) out of a possible 4 sessions in both groups. There were no dropouts among participants with SI; 1 non-SI participant dropped out of the SG. Homework completion in the MIT arm was also comparable between cohorts (*p*=.5), with SI participants completing the MIT homework exercises an average of 25 (SD 9.8) times, and non-SI participants 28 (SD 14.5) times, over 4 weeks, indicating a high rate of acceptability of home practice exercises.

### 3.2. Aim 2: outcomes in SI cohort (total n = 23; MIT n = 11; SG n = 12)

#### 3.2.1. Credibility changes in the SI cohort

Credibility in the SI cohort changed differently depending on which group participants were assigned. Within the SI cohort, the mean credibility rating in the SG was 6.3 (SD 1.2) before and 5.6 (SD 2.1) post-group, and in the MIT group it was 7.1 (SD 1.7) before and 7.4 (SD 1.7) post-group, indicating that the SG intervention evinced a reduction in credibility relative to MIT (*p*=.01). However, there was no significant difference in credibility at 4-month follow-up (*p*=.2).

#### 3.2.2. Treatment outcomes in the SI cohort

**Fig. 1.**
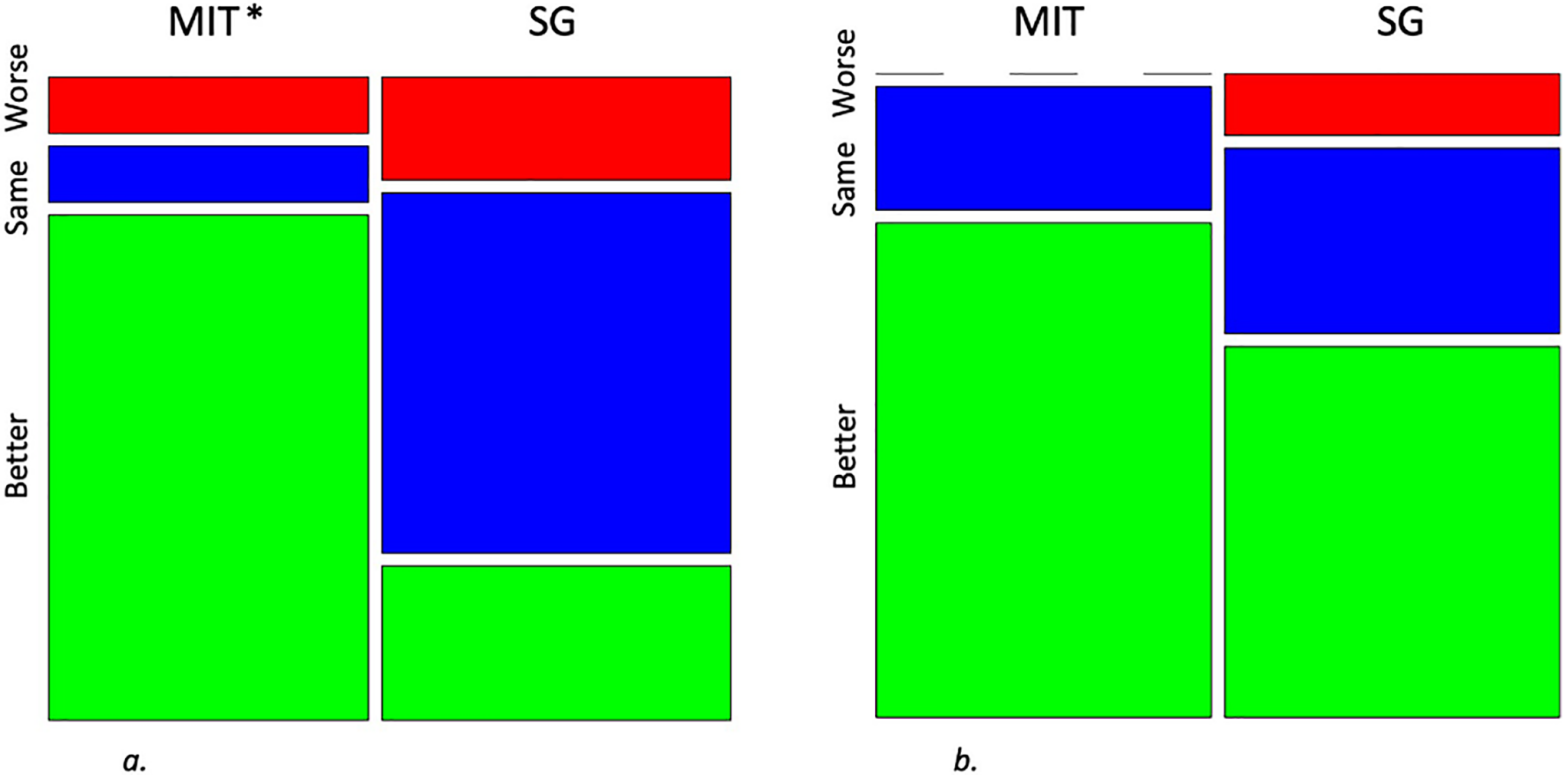
Mosaic plots showing proportions of participants in each group demonstrating worsened (red), persistent (blue), or improved (green) suicidal ideation. MIT = Mentalizing Imagery Therapy. SG = Support Group. 1a) Baseline to post-group: MIT *n* = 11, SG *n* = 12 1b) Baseline to 4-month follow up: MIT *n* = 10, SG *n* = 11. *p < .05 versus SG.

Group by Time analysis indicated that MIT was more effective than SG for participants with SI in reducing depression, anxiety, perceived stress, and increasing mindfulness (*p*≤.05 for all measures, Table 1). These effects were sustained at the 4-month follow-up. MIT did not differentially reduce caregiver burden (*p*=.09). From pre- to post-group, the MIT group demonstrated superior improvements in SI (*p*=.02, Fig. 1A). In MIT, 1 participant worsened (9 %), 1 had persistent SI (9 %), and 9 had improved SI (82 %). In SG, 2 participants worsened (17 %), 7 had persistent SI (58 %), and 3 had improved SI (25 %). By the 4-month follow up, group differences had dissipated (*p*=.63) due to further improvement in SG (Fig. 1B). In MIT at 4 months, no participants showed worsening from baseline (0 %), 2 had persistent SI (20 %), and 8 were improved (80 %). In SG at 4 months, 1 participant showed worsening (9 %), 3 had persistent SI (27 %), and 7 were improved (64 %).

#### 3.2.3. Time course of changes in the SI cohort

Paired, within-group *t*-tests established that most measures (clinician- and self-rated depression, anxiety, stress, and mindfulness) were significantly improved between baseline and post-group for MIT participants (*n* = 11) (*p*<.05 for all), but not caregiver burden (Supplementary Table 4). However, in SG (*n* = 12), only anxiety significantly improved from baseline to post-group (*p*=.005). At 4-month follow-up, MIT continued to evidence significant within group improvements in clinician- and self-rated depression, anxiety, and stress (*p*<.05 for all), with a trend toward continued improvement in mindfulness (*p*=.06). In SG, however, there were no significant within-group changes from baseline to 4-month follow-up.

## 4. Discussion

To our knowledge, this is the first study to specifically examine the effects of therapeutic interventions on SI in family caregivers of people living with dementia. The results demonstrating feasibility of MIT were strong, with low attrition, high caregiver attendance in weekly sessions, and good compliance with MIT homework completion. Relative improvement in credibility of therapy ratings in the MIT group indicate acceptability of the MIT protocol for this population. The control condition, psychoeducational SG, was chosen due to its high degree of therapist and peer support, and widespread community implementation for this population (Hornillos and Crespo, 2011), and therefore provided an active comparison for MIT.

At the post-group assessment, MIT demonstrated superior benefits to SG for reducing SI. Reasons for MIT’s apparently superior effects for reducing SI are unclear but might have been due to differential improvement of psychological symptoms, rather than caregiver burden. Previously, both caregiver psychological symptoms and caregiver burden have been associated with SI (Joling et al., 2018); however, caregiver burden was not higher in the SI cohort than those without SI in this (smaller) trial. Although MIT had significantly larger effects than did SG for reducing depressive symptoms and anxiety for caregivers with SI, there was no significant difference in improvement of caregiver burden. Moreover, within-group analyses did not demonstrate improvements in caregiver burden in either MIT or SG.

Although Group-by-Time effects favoring MIT for anxiety, stress, and mindfulness were not apparent until the 4-month follow-up, examination of within-group effects suggested that most of the improvement with MIT occurred from baseline to post-group. We attribute the lack of statistical significance in the primary Group by Time analysis on these measures at post-group to power limitations of the trial. Additionally, examination of symptom scores suggested that by the 4-month follow-up, participants in SG experienced a regression of psychological symptoms back toward baseline values (whereas improvement was largely maintained with MIT).

Worryingly, participants in SG in general experienced no improvement in SI and several showed signs of worsening SI over the course of treatment. Moreover, there was no significant within-group change in depression or stress, and only a transient improvement in stress at the post-group assessment (but not at 4 months). Reasons for this should be explored but might include sensitivity in this population to potential adverse effects of support groups (Roback, 2000; Gallagher-Thompson and Coon, 2007; Strauss, 2021), such as negative social comparison relative to others who appear to be managing better, negative anticipation generated from hearing stories of others whose relatives have more advanced dementia, and negative emotional contagion.

By 4-month follow-up, there was evidence for improvement in SI among most participants in SG. This improvement might have been spontaneous or due to participants in SG eventually finding other ways to ameliorate their psychological stress. Indeed, study staff recommended that participants with ongoing symptoms at the 4-week follow-up receive evidence-based treatments for depressive or anxious symptoms. However, of note, other determinants of SI, such as depressive symptoms and anxiety, demonstrated persistent benefits for MIT relative to SG. Taken together, the findings overall reflected that MIT was more beneficial than SG for patients with SI in this trial.

There are several important limitations to this study. While the parent trial was powered to detect change in depression in the overall sample, a subset of participants with SI was selected for this analysis. The small sample size, and resultant lack of power for statistical tests, increases the chance of Type II errors in the detection of significant results. Despite a large and clinically relevant effect size for reduction in self-reported depressive symptoms in MIT relative to SG, the Group by Time interaction was not significant. The lack of statistical significance despite the large effect size (and significant within-group effect) is a function of the lack of statistical power in this study. An additional limitation is that this study employed a limited measure of suicidality based on single-item questions on the HAM-D/QIDS. Future research should utilize a more comprehensive SI rating scale to detect more granular changes in SI.

Another limitation is that the control group for the study was not assigned home practice exercises, precluding attribution of superior benefits to specific MIT components (versus the overall program structure). Moreover, entry criteria specified that participants in both groups could have an ongoing practice of mindfulness or guided imagery no more than twice a week. As practice post-RCT was not measured, it is possible that both groups engaged in some form of ongoing mindfulness or imagery practice that could have yielded benefits for SI. A final limitation is that the clinician who administered the HAM-D was not blinded to study group, which could have introduced bias to these assessments. However, data from the parent RCT (*n* = 46) from which the SI cohort in this sample was drawn indicate that clinician ratings were more significantly correlated (*p*=.015) with strengthening of dorsolateral prefrontal cortex connectivity with an emotion regulation network (a putative mechanism) across groups than were self-ratings of depressive symptom improvement (*p*=.04) (Jain et al., 2022a). Clinician ratings thus might have had greater sensitivity to identify objective, neurobiologically-based symptoms of depression than self-ratings in this trial.

While the findings of this secondary analysis are promising for reducing psychological symptoms in family caregivers with SI, larger controlled trials should be conducted to validate the efficacy and durability of MIT in order to confirm its role in clinical practice. Furthermore, as noted above, caregiver burden did not improve in either group. Further MIT programs might help to reduce caregiver burden by adding additional features, such as a peer support chat to increase social support and emotional burden between group meetings, lengthening the course of MIT, or coupling MIT with caregiver skills training. These strategies are being trialed in ongoing studies (e.g. Jain et al., 2022b). Given its extraordinarily high prevalence, pleiotropic causes, and potentially catastrophic outcomes, we suggest that caregiver SI should be routinely studied in clinical trials of supportive interventions for this population.

## Supplementary Material

1

## Figures and Tables

**Table 1:** Clinical outcomes between groups in the SI Cohort (*N* = 23).

	Time point	MIT (*n* = 11) Mean [sd]	SG (*n* = 12) Mean [sd]	*d*	*p*
HAM-D	Screening	13.0 [6.6]	13.9 [5.5]		
	Post-group	6.1 [4.6]	11.8 [6.4]	−0.8	0.02
	4 months	6.1 [6.7]	11.6 [4.2]	−0.8	0.01
QIDS	Screening	12.1 [6.0]	10.2 [4.2]		
	Post-group	6.6 [2.5]	9.1 [4.5]	−1.0	0.1
	4 months	7.0 [4.3]	9.9 [4.8]	−1.1	0.06
STAI	Screening	51.2 [13.3]	49.8 [10.5]		
	Post-group	39.1 [11.8]	45.1 [11.8]	−0.6	0.07
	4 months	39.0 [11.6]	47.1 [10.7]	−1.1	0.05
PSS	Screening	24.4 [7.7]	22.2 [6.5]		
	Post-group	18.2 [4.2]	20.8 [5.4]	−1.0	0.1
	4 months	16.6 [5.8]	20.3 [7.7]	−0.8	0.05
CBS	Screening	50.0 [13.2]	45.0 [16.1]		
	Post-group	43.6 [12.2]	42.7 [14.3]	−0.3	0.9
	4 months	40.2 [10.0]	35.0 [11.8]	0.3	0.6
FFMQ	Screening	129.2 [22.3]	128.1 [25.4]		
	Post-group	141.5 [16.9]	133.8 [22.1]	0.5	0.09
	4 months	147.3 [20.9]	124.9 [22.5]	0.7	0.01

Notes. *d*=Cohen’s *d* for difference between groups in change; MIT=mentalizing imagery therapy; sd=standard deviation; SG=support group; SI=suicidal ideation. Assessments [range of scores]: CBS=Caregiver Burden Scale [0–88]; FFMQ=Five Facet Mindfulness Questionnaire [39–195]; HAM-*D*=Hamilton Depression Rating Scale [0–52]; PSS=Perceived Stress Scale [0–40]; QIDS=Quick Inventory of Depressive Symptomology – Self-Report [0–27]; STAI=State Trait Anxiety Inventory [20–80].

## Data Availability

Data from this trial will be made available upon reasonable request by contacting the PI (FAJ).
