# Supplementary material for: Family dementia caregivers with suicidal ideation improve with mentalizing imagery therapy: Results from a pilot study
Source: J Affect Disord Rep. Author manuscript; Available in PMC 2024 May 10. (PMC11086673; doi:10.1016/j.jadr.2024.100721)
Supplement: 1 [file NIHMS1989649-supplement-1.docx]

Supplementary Material

**Supplementary Table 1.** Congruency of SI assessments across QIDS and HAM-D.

|  | Baseline SI | Δ SI post-group | Δ SI 4-month follow-up |
| --- | --- | --- | --- |
| QIDS only | 6 | 3 | 2 |
| HAM-D only | 13 | 9 | 11 |
| Both | 4 | 3 | 3 |

SI = suicidal ideation. The “Baseline SI” column shows the numbers of assessments at baseline which demonstrate SI on one or both measures. The third and fourth columns show which measure (or if both measures) detected change at that timepoint. Assessments [range of scores]: HAM-D=Hamilton Depression Rating Scale [0-52]; QIDS=Quick Inventory of Depressive Symptomology – Self-Report [0-27].

**Supplementary Table 2.** Demographic and clinical characteristics at baseline.

|  | **SI (n=23)** | **Non-SI (n=23)** | **p** |
| --- | --- | --- | --- |
| **Age** (mean [sd]) | 63 [8.8] | 63.9 [10.1] | 1 |
| **Sex** (female %) | 87.0 | 69.6 | 0.3 |
| **Race** |  |  | 0.9 |
| White/Caucasian (%) | 69.6 | 69.6 |  |
| Black/African American (%) | 13.0 | 4.3 |  |
| Asian (%) | 8.7 | 13.0 |  |
| American Indian/Alaskan Native (%) | 0 | 4.3 |  |
| More than one race (%) | 8.7 | 8.7 |  |
| **Ethnicity –** Hispanic (%) | 4.5 | 8.7 | 1 |
| **Marital Status –** Married/partnered as  if married (%) | 56.5 | 70.0 | 0.3 |
| **Relative with dementia** |  |  | 0.3 |
| Parent (%) | 30.4 | 39.2 |  |
| Spouse/Partner (%) | 43.5 | 52.2 |  |
| More than one relative (%) | 13.0 | 0 |  |
| Other (%) | 13.0 | 8.7 |  |
|  |  |  |  |
| **Current antidepressant**  **medications (%)** | 34.8 | 13.0 | .2 |
| **Current individual therapy (%)** | 13.0 | 17.4 | .7 |
|  |  |  |  |
| **Clinician-rated depression** (mean [sd]) | 13.6 [5.8] | 6.4 [4.8] | **0.0001** |
| **Self-rated depression** (mean [sd]) | 10.7 [5.0] | 7.0 [2.9] | **0.005** |
| **Anxiety** (mean [sd]) | 50.5 [11.6] | 41.2 [10.7] | **0.004** |
| **Stress** (mean [sd]) | 23.5 [6.9] | 18.9 [6.2] | **0.02** |
| **Caregiver burden** (mean [sd]) | 48.0 [14.0] | 43.2 [15.6] | 0.2 |
| **Mindfulness** (mean [sd]) | 129.3 [23.1] | 135.2 [16.7] | 0.3 |

*Note.* SI = suicidal ideation; sd = standard deviation

**Supplementary Table 3.** Demographic and clinical characteristics of participants with suicidal ideation by group

|  | **MIT (n=11)** | **SG (n=12)** | **p** |
| --- | --- | --- | --- |
| **Age** (mean [sd]) | 61.1 [8.0] | 64.8 [9.4] | 0.3 |
| **Sex** (female %) | 100.0 | 75.0 | 0.2 |
| **Race** |  |  | 0.5 |
| White/Caucasian (%) | 63.6 | 75 |  |
| Black/African American (%) | 18.2 | 8.3 |  |
| Asian (%) | 9.1 | 8.3 |  |
| American Indian/Alaskan Native (%) | 0 | 0 |  |
| More than one race (%) | 9.1 | 8.3 |  |
| **Ethnicity –** Hispanic (%) | 9 | 0 | 1 |
| **Marital Status –** Married/partnered as  if married (%) | 54.6 | 58.3 | 1 |
| **Relative with dementia** |  |  | 0.3 |
| Parent (%) | 45.5 | 16.7 |  |
| Spouse/Partner (%) | 45.5 | 41.7 |  |
| More than one relative (%) | 0.0 | 25.0 |  |
| Other (%) | 9.1 | 16.7 |  |
|  |  |  |  |
| **Current antidepressant**  **Medications (%)** | 36.4 | 33.3 | 1 |
| **Current individual therapy (%)** | 27.3 | 0.0 | .09 |
|  |  |  |  |

*Note.* MIT = Mentalizing imagery therapy; sd = standard deviation; SG = support group

**Supplementary Table 4.** Within group changes from pre to post-group, and pre to 4-month follow-up

|  |  | Δ Pre to post-group | Cohen's d pre to post-group | *p*-value  pre to post-group | Δ Pre to 4-month FU | Cohen's *d* pre to 4-months | *p*-value  pre to 4-months |
| --- | --- | --- | --- | --- | --- | --- | --- |
| **HAM-D** |  |  |  |  |  |  |  |
|  | MIT | -6.9 [5.7] | -1.2 | **0.002** | -6.7 [7.0] | -1 | **0.02** |
|  | SG | -2.2 [4.9] | -0.4 | 0.2 | -1.3 [5.0] | -0.3 | 0.4 |
| **QIDS** |  |  |  |  |  |  |  |
|  | MIT | -5.5 [4.4] | -1.3 | **0.002** | -5 [4.2] | -1.2 | **0.004** |
|  | SG | -1.1 [3.8] | -0.3 | 0.4 | -0.3 [3.1] | -0.1 | 0.8 |
| **STAI** |  |  |  |  |  |  |  |
|  | MIT | -12.1 [15.3] | -0.8 | **0.02** | -10.9 [8.0] | -1.4 | **0.004** |
|  | SG | -5.3 [4.9] | -1.1 | **0.005** | -2.3 [4.5] | -0.5 | 0.1 |
| **PSS** |  |  |  |  |  |  |  |
|  | MIT | -6.2 [4.4] | -1.4 | **0.001** | -6.9 [7.4] | -0.9 | **0.02** |
|  | SG | -1.5 [4.2] | -0.4 | 0.2 | -0.8 [6.8] | -0.1 | 0.7 |
| **CBS** |  |  |  |  |  |  |  |
|  | MIT | -6.3 [10.0] | -0.6 | 0.06 | -4.8 [9.5] | -0.5 | 0.2 |
|  | SG | -3.2 [9.8] | -0.3 | 0.3 | -7.4 [11.1] | -0.7 | 0.08 |
| **FFMQ** |  |  |  |  |  |  |  |
|  | MIT | 12.3 [11.4] | 1.1 | **0.005** | 12.8 [17.6] | 0.7 | 0.06 |
|  | SG | 5.9 [12.7] | 0.5 | 0.2 | 1.4 [14.6] | 0.1 | 0.8 |

*d*=Cohen’s *d* for difference between groups in change; MIT=mentalizing imagery therapy; sd=standard deviation; SG=support group; SI=suicidal ideation. Assessments [range of scores]: CBS=Caregiver Burden Scale [0-88]; FFMQ=Five Facet Mindfulness Questionnaire [39-195]; HAM-D=Hamilton Depression Rating Scale [0-52]; PSS=Perceived Stress Scale [0-40]; QIDS=Quick Inventory of Depressive Symptomology – Self-Report [0-27]; STAI=State Trait Anxiety Inventory [20-80].
